# Dental erosion caused by Granny Smith apples: An evidence‐based case report and 1‐year follow‐up

**DOI:** 10.1002/ccr3.1702

**Published:** 2018-07-09

**Authors:** Entessar Z. Al Anazi

**Affiliations:** ^1^ General practioner in private clinic Riyadh Saudi Arabia

**Keywords:** Granny Smith Apples, Tooth Erosion, Preventive Management

## Abstract

Apples, if indiscriminately consumed without appropriate preventive measures to check erosion, can cause accelerated wear of the teeth.Dentists must be fully aware of the intrinsic and extrinsic factors that cause erosive tooth wear. Management strategy for tooth erosion depends on the severity of erosion, esthetics, function, and patient's preference.

## 1 INTRODUCTION

Every individual is unique, so dental treatment requires a certain degree of clinical artistry, but there are certain basic principles that are common to every case. In recent years, dentistry is increasingly being considered as a field of healthcare incorporating science as well as art. It is an established science as its fundamentals are derived from the scientific principles of research, while it is also an art because science cannot always address all the given variables in a particular clinical scenario; hence, it draws from the expertise and personal experiences of the practitioner. The basis of meaningful dental care is therefore provided by a sound understanding of common scientific principles based on research and good clinical observations and deliverance. Consequently, evidence‐based dentistry steps can be utilized to address both these building blocks of dental care. David Sackett laid the foundation of evidence‐based practice by defining it as “integrating individual clinical expertise with the best available external clinical evidence from systemic research.”^1^

Dental erosion is clinically defined as the progressive and irreversible loss of dental hard tissue caused by a chemical process of acid dissolution that does not involve bacteria.^2^ It is believed to be caused by extrinsic and intrinsic acids as well as an interplay of physiologic factors such as salivary composition and flow rate, buffering capacity, tooth composition, dental and soft tissue anatomy.^3^ The Granny Smith apple is an acidic, crisp, green apple with a unique flavor and aroma. It has a pH of 3.2, far more acidic than the pH of 5.5 that is universally acknowledged to be the threshold at which demineralization of the calcific portions of the tooth begins.^4^, ^5^

This case report describes a patient who presented with severe dental erosion on the facial and cervical aspects of the teeth. Evidence‐based methods were used to establish the causative agent as well as to formulate a treatment strategy. Although the patient rejected the management strategy presented to him, this case is academically interesting as it establishes the causative agent to be the Granny Smith apple, which is widely consumed but not reported in the literature as yet. This report increases clinicians’ awareness of the harmful acidic effects of Granny Smith apples as well as adding to the existing literature regarding extrinsic causes of dental erosion.

## 2 CASE DESCRIPTION

**Figure 1:**
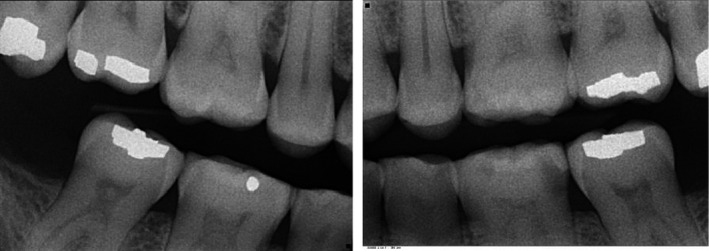
Preoperative bite‐wing radiographs

**Figure 2:**
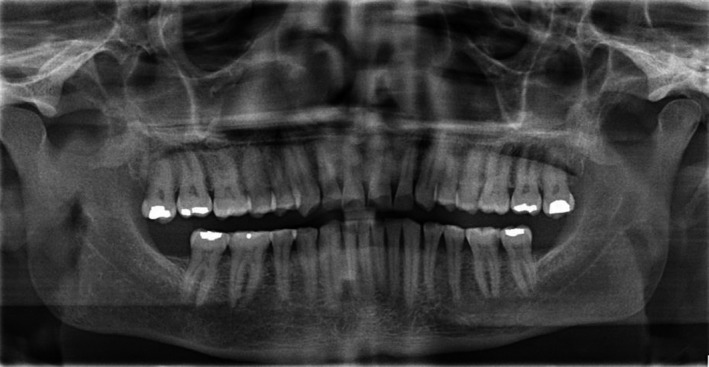
Preoperative panoramic radiograph

**Figure 3:**
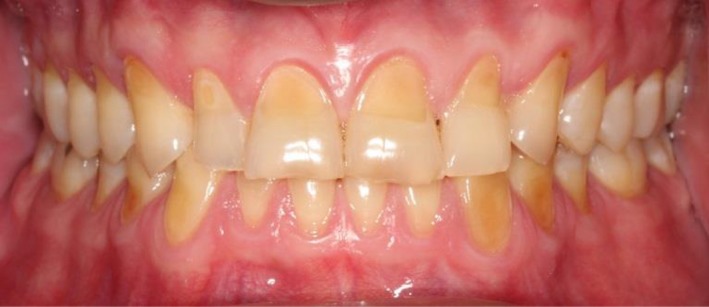
Intraoral photographs showing erosion on the cervical and facial aspects of the teeth

**Figure 4:**
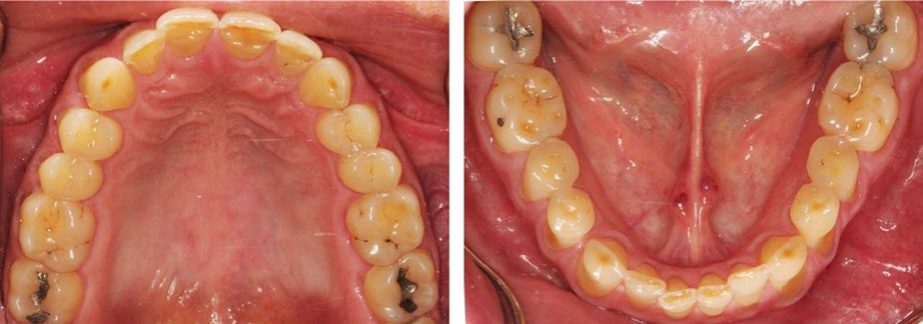
Intraoral photograph showing erosion on the incisal and occlusal aspects of the teeth

A 48‐year‐old male patient attended the clinic complaining of pain in the gum behind the lower second molar. The consent form was obtained from the patient to use clinical information and photographic material of the treatment. The patient did not report any significant medical history, except for a dental history of amalgam fillings 15 years ago. The patient brushed his teeth once daily using a medium bristled brush and a horizontal tooth brushing technique. Upon examination of the oral cavity, the upper third molar opposing the site of the pain appeared to be supra‐erupted, forming abnormal contact with the soft tissue. Radiographic examination (bitewing radiographs in Figure 1 and panoramic radiographs in Figure 2) confirmed the clinical findings. The supra‐erupted upper third molar that formed abnormal contact with the lower gum was determined as the cause of pain in the area. The oral examination also revealed generalized erosion on the facial, cervical (Figure 3), incisal and occlusal (Figure 4) surfaces of the teeth. Heat and cold sensitivity tests revealed normal results indicating that the patient experienced no dentin hypersensitivity. The medical history did not reveal usual suspected causes of erosion.

**Figure 5:**
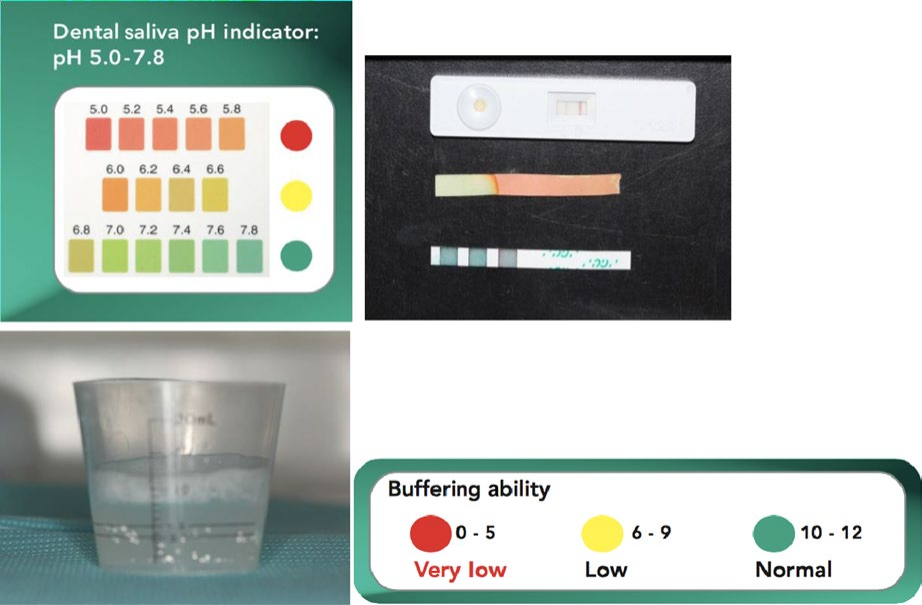
Salivary tests for quantity and pH

The patient was asked to make a diet log for 1 week until the next appointment to determine the extrinsic source of erosion as intrinsic factors were ruled out due to negative medical history (Table 1). Salivary tests were carried out to measure salivary flow, pH, and buffering capacity (Figure 5), revealing normal salivary flow with a decrease in salivary pH and buffering capacity. On examining the diet log at the next appointment, the only dietary agent that was consistently consumed by the patient that had the potential to cause such erosion was Granny Smith apples, which the patient consumed 3‐4 for lunch for the last 10 years.

**Table 1:** Diet log (1 wk)

	Breakfast	Lunch	Dinner	Snacks between meals
Sunday	Cappuccino, Cheese sandwich	Four green apples	Grilled chicken sandwich	Corn in a cup
Monday	Coffee, egg sandwich	Caesar salad, three green apples	Rice and roasted lamb, Laban	Chocolate
Tuesday	Coffee, croissant	Four green apples	Rice and grilled chicken, Laban	‐
Wednesday	Coffee, Cheese sandwich	Four green apples	Rice and lamb, lamb soup	‐
Thursday	Coffee, Cheese sandwich	Four green apples	Pizza, garlic bread	Popcorn, Ice‐cream
Friday	Scrambled egg, bread, tea	Rice, three green apples	Beef Steak, mashed potato	‐
Saturday	Bean,bread, tea	Rice, Grilled chicken, Laban (buttermilk), three green apples	Grilled chicken and rice, Salad	French fries

## 3 LITERATURE SEARCH

While following the evidence‐based strategy to deliver the patient care, this present case followed the standard five‐step approach^6^ to draft an evidence‐based treatment strategy suitable for the needs of the patient. This clinical scenario was converted to the PICO format wherein P refers to the Problem, I refers to the Intervention, C refers to Comparison, and O is the Outcome of the treatment.^7^, ^8^ Keeping this in mind, the PICO question that resulted from this clinical scenario was “Whether the teeth with dental erosion (P) should be managed with preventive care (I) or restorative treatment (C) and what will the expected prognosis be (O)?”

With this question in mind, a standard literature search was conducted in PubMed and Google Scholar using keywords for each component of the PICO format as illustrated in Table 2. The search terms were converted into Medical Subject Headings (MeSH) terms where possible. MeSH refers to the controlled vocabulary thesaurus that is developed by the National Library of Medicine that gives uniformity and consistency to the indexing and cataloging of biomedical literature.^9^ The search revealed one meta‐analysis, two systematic reviews, eight review articles, two in vitro case‐control studies, and two in vitro case studies pertaining to the search terms entered (Table 3).

**Table 2:** Search terms or keywords used during literature search

Pico question	Search terms
P	Dental Erosion or Tooth Erosion (MeSH), granny smith apple
I	Preventive therapy of tooth erosion, prophylaxis
C	Restorative treatment, dental erosion management
O	The above search terms revealed what was required to ascertain the outcome of the interventions

**Table 3:** Literature search

Source of literature	Relevant studies
PubMed (http://www.pubmed.gov) and Google Scholar (https://scholar.google.com)	1 Meta‐analysis Li H, Zou Y, Ding G (2012) Dietary factors associated with dental erosion: a meta‐analysis. PLoS ONE. 2012 Aug; 7(8): e42626.
	2 Systematic Reviews Joshi M, Joshi N, Kathariya R, Angadi P, Raikar S. Techniques to evaluate dental erosion: a systematic review of literature. Journal of Clinical and Diagnostic Research : JCDR. 2016;10(10):ZE01‐ZE07 Amaechi BT, Higham SM. Dental erosion: possible approaches to prevention and control. Journal of Dentistry. 2005 Mar;33(3):243‐52
	7 Review Articles Irilfeld T. Prevention of progression of dental erosion by professional and individual prophylactic measures. European Journal of Oral Sciences. 1996; 104: 215‐22 Davies SJ, Gray RJ, Qualtrough AJ. Management of tooth surface loss. British Dental Journal. 2002 Jan 12;192(1):11‐6, 19‐23 Johansson A‐K, Omar R, Carlson GE, Johansson A. Dental erosion and its growing importance in clinical practice: from past to present. International Journal of Dentistry. 2012;2012:632907 Wang X, Lussi A. Assessment and management of dental erosion. Dental Clinics of North America. 2010 Jul;54(3):565‐78 Smith BG, Bartlett DW, Robb ND. Prevalence, etiology and management of tooth wear in the United Kingdom. J Prosthet Dent. 1997 Oct;78(4):367‐72. Yip HK, Smales RJ, Kaidonis JA. Management of tooth tissue loss from erosion. Quintessence Int. 2002 Jul‐Aug;33(7):516‐20. Ganss C, Lussi A, Schlueter N. Dental erosion as oral disease. Insights in etiological factors and pathomechanisms, and current strategies for prevention and therapy.Am J Dent. 2012 Dec;25(6):351‐64. Bartlett D, Ganss C, Lussi A. Basic Erosive Wear Examination (BEWE): a new scoring system for scientific and clinical needs.Clin Oral Investig. 2008 Mar;12Suppl 1:S65‐8.
	2 in‐vitro Case‐Control Studies Willershausen B, Callaway A, Azrak B, Duschner H. Influence of apple juice on human enamel surfaces of the first and second dentition. Eur J Med Res. 2008 July;13:349‐354 Poggio C, Gulino C, Mirando M, Colombo M, Pietrocola G. Preventive effects of different protective agents on dentin erosion: An in vitro investigation. J ClinExp Dent.2017;9(1):e7‐e12.
	2 in‐vitro Case Studies De Moraes MDR, Carneiro JRM, Passos VF, Santiago SL. Effect of green tea as a protective measure against dental erosion in coronary dentin. Braz Oral Res. 2016;30:e13 Badr SBY, Ibrahim MA. Protective effect of three different fluoride pretreatments on artificially induced dental erosion in primary and permanent teeth. Journal of American Science. 2010;6(11)

**Figure 6:**
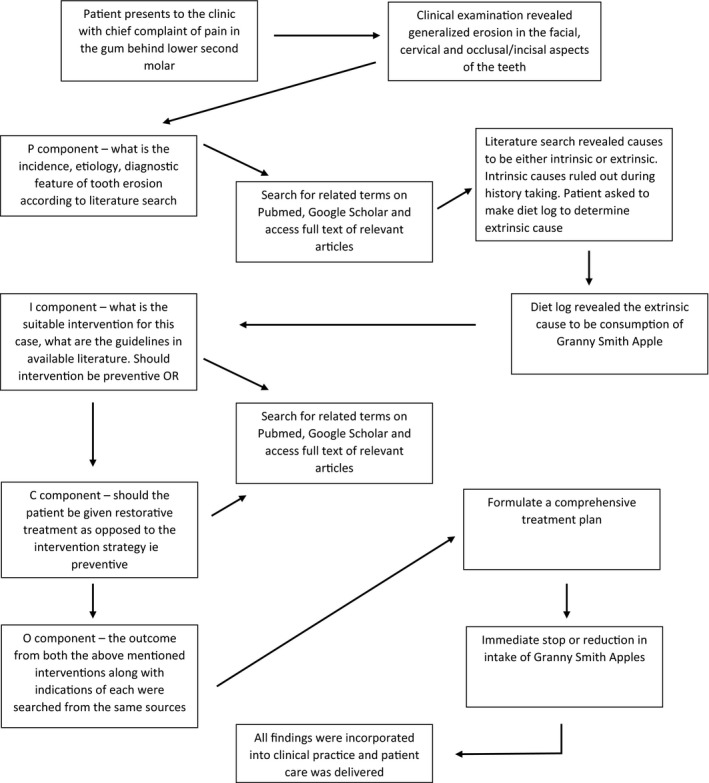
Evidence‐based process used for treatment planning of the present case

Dental erosion is a widely researched subject and is extensively reported in the literature. According to the Bulletin of the World Health Organization,^10^ it affects 8%‐13% of the adult population in developing countries and is caused by consumption of acidic beverages. An evidence‐based approach was used through the diagnosis and treatment planning stage as depicted in Figure 6. The causative agent was established at this stage to be the Granny Smith apples after examining the diet log submitted by the patient. The next step was the formulation of a management strategy to contain the erosive process. Bartlett et al^11^ developed a scoring system for erosive wear of the teeth, where the tooth surface most severely affected by dental erosion is in each sextant recorded based on a four‐point score system. The score is then added to obtain a total which is matched with risk levels to guide the management strategy. This is called the Basic Erosive Wear Examination (BEWE) and it is a simple, reproducible, and transferable scoring system for recording clinical findings and for assisting in the decision making process for the management of erosive tooth wear.^11^ The BEWE assigns the following scores based on the severity of erosion^11^: 0—no erosive tooth wear; 1—initial loss of surface texture; 2—distinct defect, hard tissue loss which involves <50% of the surface area (dentin involved); 3—hard tissue loss greater than 50% of the tooth surface.

Table 4 depicts the management strategies recommended by Bartlett et al based on the cumulative score. According to Wang and Lussi,^12^ restorative treatment is warranted in case the affected surfaces are compromised to the degree that: the structural integrity of the tooth is threatened; the exposed dentin is hypersensitive; erosive defect is esthetically unacceptable to the patient; or the defect is so deep that it may result in pulpal exposure. Davies et al^11^ further recommend that active treatment with restorations is only necessary in cases of noncarious tooth surface loss, when there is concern regarding one or more of the following factors: esthetics, sensitivity, function, or space loss in vertical dimension. Based on these recommendations and information obtained from the patient, a treatment plan was formulated.

**Table 4:** Risk levels as a guide to clinical management (adapted from Bartlett et al)^11^

Risk Level	Cumulative Score of all Sextants	Management
None	Less than or equal to 2	Routine maintenance and observation Repeat at 3‐year intervals.
Low	Between 3 and 8	Oral hygiene and dietary assessment and advice, routine maintenance, and observation Repeat at 2‐year intervals
Medium	Between 9 and 13	Oral hygiene and dietary assessment, and advice, identify the main etiological factor(s) for tissue loss and develop strategies to eliminate respective impacts. Consider fluoridation measures or other strategies to increase the resistance of tooth surfaces Ideally, avoid the placement of restorations and monitor erosive wear with study casts, photographs, or silicone impressions. Repeat at 6‐ to 12‐month intervals.
High	14 and above	Oral hygiene and dietary assessment, and advice, identify the main aetiological factor(s) for tissue loss and develop strategies to eliminate respective impacts. Consider fluoridation measures or other strategies to increase the resistance of tooth surfaces. Ideally, avoid restorations and monitor tooth wear with study casts, photographs, or silicone impressions. In particular, in cases of severe progression consider special care that may involve restorations. Repeat at 6‐ to 12‐month intervals.

## 4 EVIDENCE‐BASED TREATMENT PLAN AND MANAGEMENT

**Figure 7:**
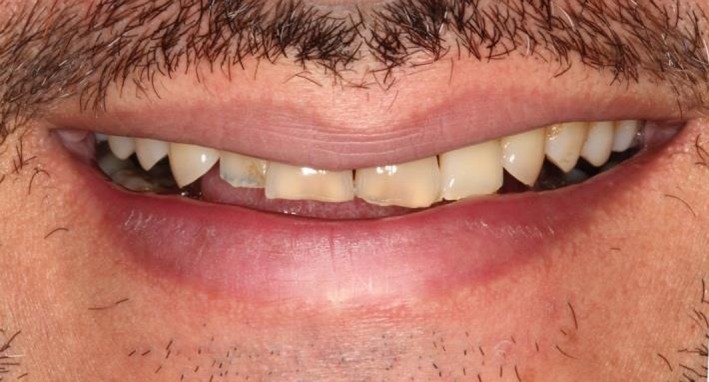
Patient's teeth hardly visible upon smiling

At the next appointment, the patient's BEWE score was recorded (Table 5) and risk levels were assessed. Based on the examination, the patient fell under low risk which did not require active/restorative treatment. Furthermore, the absence of sensitivity and patient's satisfaction with the appearance and function of his teeth did not require any restorative treatment because the teeth were not visible upon smiling (Figure 6). The supra‐erupted third molars were extracted (Figure 7A), and the proximal surface of the lower left first molar was restored using composite resin (Figure 7B). Topical application of fluoride was performed on all the teeth (Figure 7C). The patient was counseled with regard to his diet and oral hygiene to reduce his intake of the acidic agent, that is, the Granny Smith apple, to follow up any exposure to an acidic agent with milk or cheese, use a straw to drink any acidic beverage, use a medium to soft bristled toothbrush, and avoid brushing too hard.

**Figure 8:**
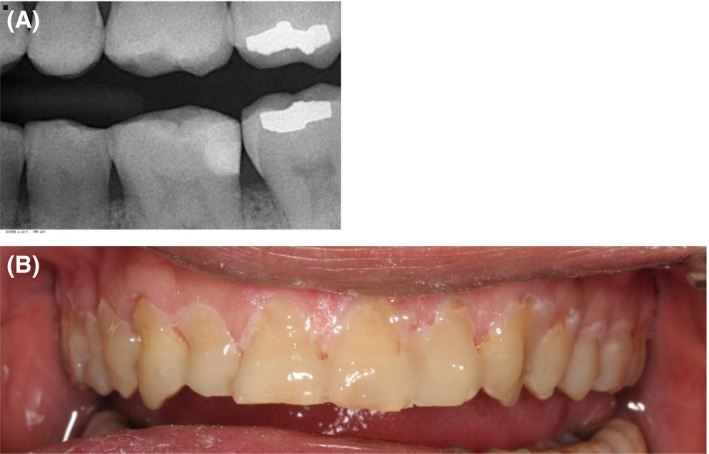
A, Postoperative bite‐wing radiograph showing proximal restoration. B, Fluoride application on the teeth

**Table 5:** Pre‐treatment Basic Erosive Wear Examination (BEWE) score of the patient

Sextant	Score
Sextant 1 (17‐14)	1
Sextant 2 (13‐23)	2
Sextant 3 (24‐27)	1
Sextant 4 (37‐35)	1
Sextant 5 (34‐44)	2
Sextant 6 (45‐47)	1
Total	8

## 5 FOLLOW‐UP AND PROGNOSIS

**Figure 9:**
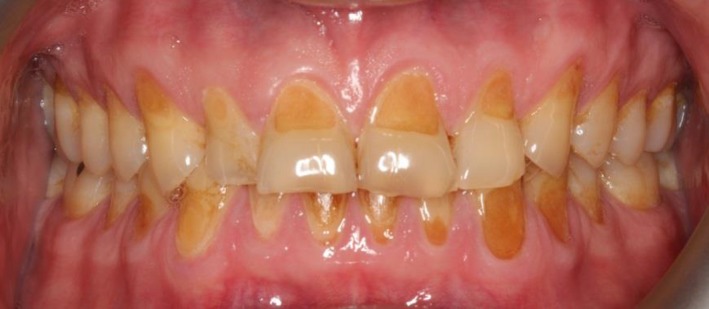
Post‐treatment photograph at follow‐up after 1 year showing extrinsic staining of teeth

**Figure 10:**
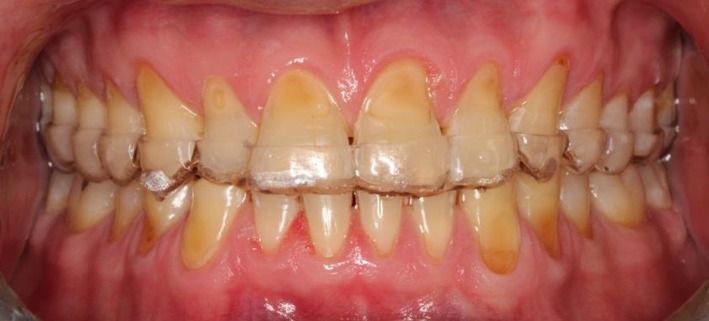
After dental prophylaxis and insertion of night guard at the 1‐year follow‐up appointment

The patient was recalled for a follow‐up visit after 12 months, the examination revealed extrinsic staining on the teeth (Figure 9), indicating a reduced intake of the erosive agent, that is, Granny Smith apple. Discussion with the patient confirmed that he had reduced his consumption of apples and was following the diet and oral hygiene advice. Dental prophylaxis was performed on the extrinsically stained teeth, and the patient was prescribed a night guard (Figure 10). The teeth were scored again using the BEWE index and assessed for risk levels (Table 6). The examination revealed a consistent total score and risk level highlighting the positive maintenance of the erosive condition.

**Table 6:** BEWE score of the patient at 1 year follow‐up appointment

Sextant	Score
Sextant 1 (17‐14)	1
Sextant 2 (13‐23)	2
Sextant 3 (24‐27)	1
Sextant 4 (37‐35)	1
Sextant 5 (34‐44)	2
Sextant 6 (45‐47)	1
Total	8

## 6 CONCLUSION

The relationship between certain dietary factors and dental erosion is well established. Apples, if indiscriminately consumed without appropriate preventive measures to check erosion, can cause accelerated wear of the teeth. As in the present case, dental erosion is usually a chance encounter in the clinic and seldom presented by the patient unless there is some debilitation involved. Severe erosion can lead to dentin hypersensitivity, pulpal involvement, unpleasant appearance, as well as loss of function in advanced cases. The dentist must be familiar with the identification, causes, and management strategies pertaining to noncarious tooth wear in general and dental erosion in particular. Evidence‐based decision making is crucial for delivering quality dental care, equipping the practitioner with the necessary theoretical knowledge and allowing them to make decisions based on the established guidelines and treatment protocols as well as on the latest advancements in techniques and practice methodologies.

## CONFLICT OF INTEREST

The author declares that there is no conflict of interest regarding the publication of this manuscript.

## AUTHORSHIP

Al Anazi EZ: diagnosed the case, patient management, took photos and wrote the manuscript.
